# Personality Predictors of Yips and Choking Susceptibility

**DOI:** 10.3389/fpsyg.2019.02784

**Published:** 2020-01-21

**Authors:** Philip Clarke, David Sheffield, Sally Akehurst

**Affiliations:** Human Sciences Research Centre, University of Derby, Derby, United Kingdom

**Keywords:** yips, choking, paradoxical performance, performance under pressure, stress, personality, individual characteristics

## Abstract

The ability to perform under heightened levels of pressures is one of the largest discriminators of those who achieve success in competition and those who do not. There are several phenomena associated with breakdowns in an athlete’s performance in a high-pressure environment, collectively known as paradoxical performances. The two most prevalent and researched forms of paradoxical performance are the yips and choking. The aim of the current study is to investigate a range of psychological traits (fear of negative evaluation, individual differences, anxiety sensitivity, self-consciousness, perfectionistic self-presentation, and perfectionism) and their ability to predict susceptibility to choking and the yips in an experienced athlete sample. 155 athletes (Golfers *n* = 86; Archers *n* = 69) completed six trait measures and a self-report measure of yips or choking experience. The prevalence rate for choking and yips in both archers and golfers was 67.7 and 39.4%, respectively. A 2 × 2 × 2 MANOVA and discriminant function analysis revealed that a combination of 11 variables correctly classified 71% of choking and non-choking participants. Furthermore, analysis confirmed that a combination of four variables correctly classified 69% of the yips and non-yips affected participants. In this first study to examine both paradoxical performances simultaneously, these findings revealed that for the yips, all predictors stemmed from social sources (i.e., perfectionistic self-presentation), whereas choking was associated with anxiety and perfectionism, as well as social traits. This important distinction identified here should now be tested to understand the role of these traits as development or consequential factors of choking and the yips.

## Introduction

In sport, the difference between success and failure depends on an individual’s ability to successfully execute motor skills under heightened levels of pressure. Research over the last three decades has investigated performance under pressure and various phenomena associated with athletes who struggle to perform when it matters most (Hill et al., 2010; Lobinger et al., 2014). These phenomena have been identified as paradoxical performances, whereby “*the occurrence of inferior performance despite striving and incentives for superior performance*” (Baumeister and Showers, 1986; p. 288). Two of the most common and closely linked forms of paradoxical performance are the yips and choking (Lobinger et al., 2014).

**FIGURE 1:**
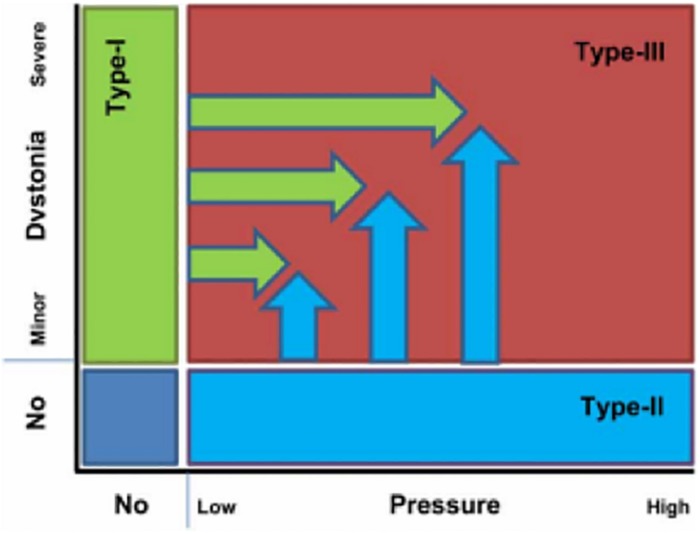
Clarke et al. (2015) Yips Classification Model.

The yips have been defined as “*a psycho-neuromuscular impediment affecting the execution of fine motor skills during sporting performance*” (Clarke et al., 2015, p. 177). Clarke et al. (2015) expanded on Smith et al.’s (2000) model to propose a two-dimensional yips model (see Figure 1). The updated model includes athletes who predominantly experience physical symptoms of the yips as type-I (focal-dystonia); those who predominantly experience psychological symptoms of the yips as type-II (performance anxiety); and those who experience both psychological and physical symptoms of the yips as type-III (focal-dystonia and performance anxiety). Both type-II and type-III yips include aspects of performance anxiety-related symptoms. Choking is an extreme outcome of the anxiety and performance relationship (Baumeister, 1984) and has been suggested as the best explanation for the psychological components of the yips (Bawden and Maynard, 2001). This is supported by qualitative accounts where yips-affected athletes exhibited similar characteristics to a severe form of choking, for example, heightened self-consciousness (e.g., Bennett et al., 2015). However, a recent review highlighted the lack of clarity between what constitutes a yip or a choke (Lobinger et al., 2014) in the literature. Clark et al. (2005) reported one key difference between the yips and choking, in that chokers are still able to make rational decisions and choose the correct path for successful performance, but performance is hindered by psychological factors. By contrast, the yips are characterized by the uncontrollability of physical movement, which can be worsened by psychological distress. This proposal would suggest that yips are not caused by anxiety factors but can be affected by them. However, both the yips and severe choking share several similarities in the psychological symptoms experienced (e.g., Bennett et al., 2015). Therefore, a key difference in choking and particularly type-II and type-III yips stems from the severity of the psychological symptoms experienced. For instance, Lobinger et al. (2014) proposed that the yips may be a conditioned reaction to many previous choking experiences or one significant emotion-laden choking experience. This was based on the observation that choking is characterized by an acute incident (i.e., one off) and the yips may represent a more chronic form of choking (Klämpfl et al., 2013a; Lobinger et al., 2014). Therefore, to gain a comprehensive understanding of the yips, it is imperative to explore the role of choking and the yips simultaneously. This will allow for a clearer understanding of the differences and similarities between the psychological factors associated with both, and thus, will be explored in the current study.

Research has recently been investigating the influence of individual differences on paradoxical performances (Roberts et al., 2013; Byrne et al., 2015; Laborde et al., 2019). Individual differences have been assessed using two approaches: type-based assessments (to categorize individuals as one type or another) or trait-based assessments (to position individuals on a linear continuum). Both approaches (type and trait based) have provided the foundation for the development of the Big-Five personality dimensions, which may not represent a specific theoretical perspective, but do provide descriptions of the most basic general dimensions upon which individuals differ (Allen et al., 2013). These dimensions include: extraversion, assessing interpersonal interactions; openness, assessing the desire to seek out new experiences; neuroticism, assessing an individual’s level of emotional instability (e.g., anxiety and self-consciousness); conscientiousness, assessing goal directed behavior and organizations; and agreeableness, which assesses social harmony and concern for cooperation. This is a widely accepted model of a personality trait structure (McCrae and Costa, 2008) that has been associated with performance in several personal, interpersonal and social domains (Oh et al., 2011; Poropat, 2011). For example, Bell (2007) reported that agreeableness, conscientiousness and openness to experience were strong predictors for team performance, demonstrating the influencing role these traits can have in the sport domain and, as such, deserve further investigation in sporting performance (Allen et al., 2013).

Recent reviews of choking (Hill et al., 2010) and the yips (Clarke et al., 2015) suggest that more research investigating the role of personality traits as potential predictors, is warranted to identify those individuals more susceptible to yips and choking. To date, limited research has assessed the role of the big-five and paradoxical performance; with only one paper, to the author’s knowledge, investigating this in relation to choking only (Byrne et al., 2015). Byrne et al. (2015) found that lower levels of neuroticism and agreeableness were associated with poor performance during social pressure, and social and time pressure. Byrne et al. (2015) suggested that this provides support for distraction theories such as the attentional control theory (ACT: Eysenck et al., 2007; Eysenck and Derakshan, 2011) whereby finite attentional resources are devoted to ruminative thoughts and thus resources are not available for task relevant stimuli. Of the limited studies to have investigated other personality traits as potential predictors of both the yips (e.g., perfectionism by Roberts et al., 2013) and choking (e.g., self-presentation by Mesagno et al., 2012), all have adopted a trait-based approach, allowing for an accurate assessment of personality test scores on a probability distribution (Allen et al., 2013), yet more research of this nature is needed to expand our understanding of the role of personality on paradoxical performance. Accordingly, the current study will adopt a trait-based approach to investigate potential predictors associated with both the yips and choking.

The predictive factor that has received the most attention in the paradoxical performance literature is anxiety (Lobinger et al., 2014). Performance anxiety has been highlighted as an important contributor to the three yips types in the two-dimensional mode (Clarke et al., 2015) and the occurrence of choking (Lobinger et al., 2014). Athletes who have high levels of trait anxiety have also been identified as being more susceptible to choking (e.g., Wilson, 2008), however, this was not the case in those who experienced the yips (e.g., Klämpfl et al., 2013a). Caution is warranted when interpreting these results in the yips study as small sample sizes were recruited that were only powered to detect large effect sizes (*n* = 24–50). Moreover, qualitative accounts of both the yips (Philippen and Lobinger, 2012; Prior and Coates, 2019) and choking (Guicciardi et al., 2010) propose that an individual’s interpretation of anxiety symptoms may be a stronger indicator of performance impact than intensity *per se* (Prior and Coates, 2019). Furthermore, a review of generalized anxiety by Newman and Llera (2011) suggested that extremely anxious individuals may be hypersensitive to changes in emotional states, which can directly influence upcoming events or performances, such as competition. Anxiety sensitivity is believed to be a stable trait-like characteristic (Schmidt et al., 1997) that relates to the degree to which an individual interprets automatic arousal as having harmful consequences (Schmidt et al., 1997) and where, cognitive misappraisal of these characteristics may have negative implications for experiencing anxiety. This supports the Directional Interpretation Hypothesis (Jones and Hanton, 2001) which suggests that individuals who perceive anxiety as facilitative experience enhanced performance, whereas individuals who experience anxiety as debilitative are more likely to experience a drop in performance. This is potentially due to ACT principles as described above (Eysenck and Derakshan, 2011). As such, a trait measure of an individual’s perception toward changes in arousal may provide important insight into the role of anxiety within paradoxical performance, and thus will be explored in the current study.

The role of traits in predicting paradoxical performance has also been explored in other performance domains where yips-like symptoms occur such as musician’s dystonia. For instance, research has reported that trait anxiety increased the likelihood of musicians being diagnosed with focal dystonia (e.g., Lehn et al., 2014). These findings support Lencer et al.’s (2009) proposal (highlighted earlier) that high levels of trait anxiety and focal dystonia both show decreased levels of cortical inhibition. Altenmüller and Jabusch (2009) have further suggested professional pressure (anxiety) and perfectionism as facilitating factors for the onset of musician’s dystonia and was also likely that of yips-affected athletes (Ioannou et al., 2018). This suggests that an understanding of psychological traits in the experience of movement disorders is a viable avenue of research. However, it is worth noting that it is unclear how these psychological characteristics contribute to dystonia symptoms, and whether they are pre-existent or psycho-reactive (Lehn et al., 2014).

Perfectionism has been identified as a potential predictor of the yips and choking, yet the literature to date has been equivocal (Klämpfl et al., 2013b; Bennett et al., 2016). Guicciardi et al. (2010) explored the experience of choking in 22 experienced golfers revealing that when the golfers set excessively high standards and goals prior to a choke, it precipitated a feeling of anxiety. Furthermore, they highlighted that athletes who partook in critical evaluation of their performance post-choke, were susceptible to experiencing chronic forms of choking, and were likely to view similar situations as threatening. Whilst in a yips sample, Roberts et al. (2013) and Bennett et al. (2016) found five perfectionistic tendencies (personal standards, organization, doubts about actions, concern over mistakes and parental criticism) were associated with yips behavior. In contrast, Klämpfl et al. (2013b) revealed no differences between any of the tendencies between those yips-affected and unaffected athletes. Although, this may be a consequence of low sample sizes (Bennett et al., 2016) and low scores for each measure reported (Sapieja et al., 2011; Roberts et al., 2013). Consequently, it is important that future research recruits adequately powered samples to allow confident conclusions to be derived avoiding type two errors.

Interestingly, perfectionism has been linked with self-presentational concerns. Sorotzkin (1985) reported that perfectionists experienced a compelling need for acceptance and admiration that manifested in a socially acceptable impression, which defends them from potential rejection, and promotes idealized social qualities. Furthermore, Leary (1992) proposed that competitive anxiety revolves around the self-presentational implications of competition (providing an ideal image). Research has indicated that individuals, who attempt to create a public image, which supports their preferred self-beliefs, will experience increased anxiety in situations where there is a chance of appraisal from both internal and external sources (Leary, 2001). Hobden and Pliner (1995) identified that perfectionists, especially those with socially prescribed anxiety, would utilize self-presentational or impression management strategies such as face saving or self-handicapping to cope effectively with socially derived impressions. However, research into paradoxical performance has yet to investigate this link, so the current study aims to provide a novel investigation of the role of self-presentational tendencies associated with perfectionism, such as individuals trying to perfect how they are viewed in public (Besser et al., 2010).

Hewitt et al. (2003) developed a perfectionistic self-presentational model that incorporated three traits of self-presentation: perfectionistic self-promotion; non-display of imperfection; and non-disclosure of imperfection. Perfectionistic self-promotion distinguishes between an individual’s pursuit of perfection in the eyes of others and a focus that involves diminishing the influence of the public perception (Higgins, 1998). Non-display of imperfection encompasses a desire to refrain from publicly displaying any imperfections or being presented in a less than perfect manner (Hewitt et al., 2003). Furthermore, non-disclosure of imperfection comprises an avoidance action, whereby an individual abstains from verbal disclosures of any perceived or personal imperfections (Hewitt et al., 2003). Flett and Hewitt (2014) reported that understanding these forms of self-presentation is particularly important when trying to understand people who perform in front of crowds. Interestingly, an understanding of these traits can provide an alternative insight into the role of social pressure and levels of self-consciousness when performing. Specifically, as public self-consciousness was highlighted as being a contributing factor to those who experienced choking (Geukes et al., 2012). Thus, this study aims to investigate the role of perfectionism, perfectionistic self-presentation and measures of self-consciousness and fear of negative evaluation (FNE) in relation to two forms of paradoxical performance.

**FIGURE 2:**
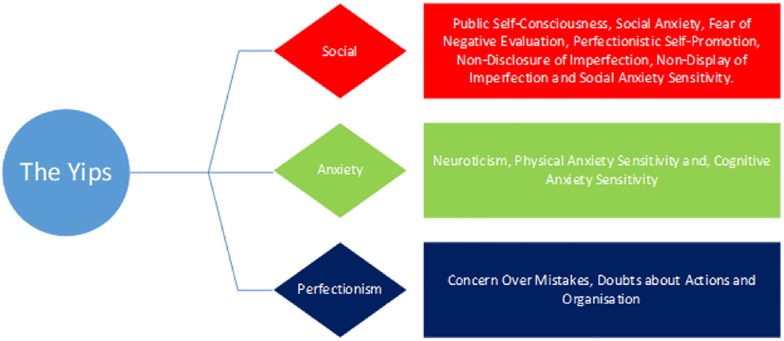
The hypothesized Yips Predictive Model (YPM).

**FIGURE 3:**
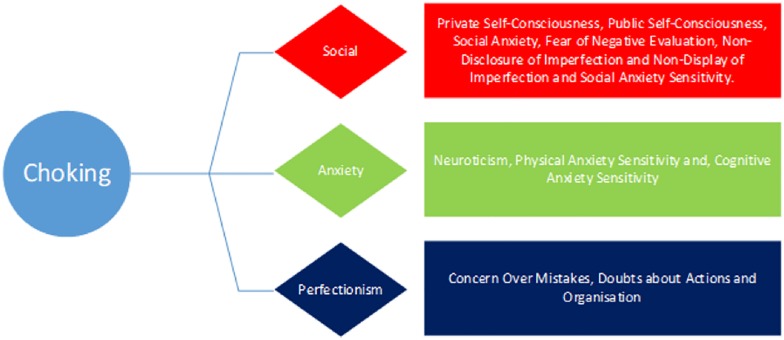
The hypothesized Choking Predictive Model (CPM).

To summarize, this study aims to investigate whether several psychological traits (FNE, individual differences, anxiety sensitivity, self-consciousness, perfectionistic self-presentation, and perfectionism) predict whether individuals are more likely to experience different forms of paradoxical performance, specifically the yips and choking. As there are 20 different variables being measured in the current study, these have been categorized based on their underlying constructs, namely anxiety, social and perfectionism. It is expected that the sources that stem from anxiety, social and perfectionism sources will be higher in those more susceptible to both choking and the yips than those who are unaffected. We propose two predictive models for both the yips (YPM) and choking (CPM). Our hypotheses for each model are illustrated in the figures below (Figures 2, 3).

## Materials and Methods

### Participants

One hundred and fifty-five (Male *n* = 78, *M*age = 43.35, SD = 14.48; Female *n* = 23, *M*age = 47.70, SD = 11.47; unknowns *n* = 54) participants volunteered to take part in this online questionnaire study; 54 participants’ gender and age were not recorded due to an issue with computer software. An *a priori* power analysis conducted in G^∗^Power revealed that 50 participants would be sufficient to detect a small to medium effect size, partial η^2^ of 0.08, assuming a power of 0.8 and alpha of 0.05. Using the findings from Roberts et al. (2013), where the effect size ranged from *d* = 0.52 to *d* = 0.035, the conservative estimate of the potential effect size in the current study was deemed appropriate, due to number of predictors, relative to previous studies (Roberts et al., 2013). Both golfers (*n* = 86) and archers (*n* = 69) were recruited as previous research has reported that the yips are particularly prevalent in both these sports (e.g., Prior and Coates, 2019). All participants were (a) aged 18 or older, and (b) either an archer who competed at county level and above, or a golfer with a handicap of 15 or below. Recruitment for the study was obtained using opportunity sampling by contacting governing bodies, using personal contacts within sport (sending emails with links to online study) and through social media (Facebook and Twitter). This research complied with The British Psychological Society’s ethical guidelines (BPS, 2013) and ethical approval was obtained from the Sport and Exercise Research Ethics Committee (Ethic approval Number: SPORTX_1314_04) at the University.

### Design

A 2 × 2 × 2 independent design was employed to explore the role of FNE, anxiety sensitivity, perfectionism, perfectionism self-presentation, self-consciousness, and individual differences between yips (yips-affected and unaffected) and choking (choking-affected and unaffected) across two sports (Golf and Archery).

### Measures

Questionnaires measured FNE, anxiety sensitivity, perfectionism, perfectionism self-presentation, self-consciousness, individual differences, and perceived control using an online survey tool, www.qualtrics.com. The Cronbach’s alpha measure for all measures were appropriate (Cronbach’s α > 0.06) apart from agreeableness, openness and non-disclosure of imperfection (Cronbach’s α < 0.06).

*Brief Fear of Negative Evaluation-II* (BFNE-II: Carleton et al., 2006) is a shorter version of the FNE questionnaire (Watson and Friend, 1969) that measures an individual’s tolerance for the possibility they may be judged despairingly or with hostility by others. The BFNE-II has acceptable psychometric properties (Carleton et al., 2006) and consists of 12 items rated on five-point Likert scales ranging from 0 (not at all characteristic of me) to 4 (extremely characteristic of me).

*Anxiety Sensitivity Index-III* (ASI-III: Taylor et al., 2007) is an 18-item version of the original ASI that measures fear of physical, cognitive and social domains of anxiety on a five-point Likert scale from 0 (very little) to 4 (very much). Six items measure fear of physical symptoms, six items measure fear of cognitive control and the final six items measure fears of social concerns.

*Multidimensional Perfectionism Scale* (FMPS: Frost et al., 1990). The shortened version of the FMPS used in the current study has good psychometric qualities. The shortened FMPS is a 22-item questionnaire that assesses five dimensions of perfectionism on a five-point Likert scale from 1 (strongly disagree) to 5 (strongly agree). The five dimensions measured included: concern over mistakes; organization; personal standards; parental pressures; and doubts about action.

*Perfectionism Self-Presentation Scale* (PSPS: Hewitt et al., 2003) is a 27-item multidimensional scale that evaluates an individual’s need to appear perfect to others on a seven-point Likert scale from 1 (disagree strongly) to 7 (agree strongly). The scale consists of three subscales: perfectionistic self-promotion which assesses the need to appear perfect to others; non-display of imperfection which assesses the need to avoid looking imperfect to others; and non-disclosure of imperfection which assesses the need to avoid revealing imperfections to others.

*Self-Consciousness Scale* (SCS, Fenignstein et al., 1975) is a 23-item questionnaire that measures dispositional self-consciousness on a five-point Likert scale from 0 (extremely uncharacteristic) to 4 (extremely characteristics). The scale consists of three subscales: private self-consciousness; public self-consciousness; and social anxiety.

*The Big-Five Inventory-10* (BFI-10: Rammstedt and John, 2007) is a shortened version of the Big-Five Inventory that consists of 44 items assessed on a five-point Likert scale from 1 (disagree strongly) to 5 (agree strongly). The BFI-10 assesses the big-five characteristics: extraversion; agreeableness; conscientious; neuroticism; and openness to experiences. The BFI-10 showed good psychometric qualities and had better test-retest reliability than other 10-item personality measures (Rammstedt and John, 2007).

*Demographics* were reported via a form created to collect data on gender, age, level of competition (school/university, club, county, national, international), handicap (for golf only), and time spent at each level. Choking demographic information was recorded via self-report that identified if the participants “*had ever experienced a dramatic drop in performance that had been out of their control*.” Yips demographic information was recorded via a self-report measure which identified if the participants “*had ever experienced the yips (golf) or target-panic (archery)*.” Those in the yips group identified yes on this scale and answered a few yips specific questions such as: severity of the yips on performance; aspect of the game affected (golf); bow affected (archery); how long they had suffered with symptoms; are they currently suffering, and when was their last experience of the yips.

### Procedure

If the participant was interested in taking part in the study they clicked on the online link that was hosted by www.qualtrics.com. Participants were then presented with the study information sheet and a series of questions regarding informed consent and the right to withdraw. Upon providing consent the participant created a unique identifying code (made up of three letters and three digits) which allowed for their data to be identified if they wished to withdraw. The six questionnaires were presented in a randomized order (BFNE, ASI-II, SCS, BFI-10, PSPS, and the FMPS), followed by the choking and yips specific questions respectively. The final debrief page provided further information about the study, it restated the right to withdraw, and provided details about sources of support for information.

### Analysis

Data was analyzed using SPSS version 25. Normality of continuous variables was confirmed by histograms and Kolmogorov–Smirnov and Shapiro–Wilk tests. To explore the differences in scores of anxiety sensitivity, FNE, perfectionism, perfectionism self-presentation, self-consciousness, and individual differences between those participants in the yips, choking and control groups, and between archery and golf, a 2 × 2 × 2 MANOVA was employed. To test which variables best predicted yips and choking behavior, Discriminant Function Analyses were conducted (Field, 2013). All tests were two-tailed with an alpha set at 0.05.

## Results

### Demographics

Most of the scales used in the current study were classed as reliable (Cronbach’s α > 0.5; George and Mallery, 2003) based on Cronbach’s Alpha test. There were issues with reliability for the subscales of agreeableness, openness, and non-disclosure of imperfection.

#### Choking

Table 1 provides the mean scores for age, handicap and experience at the highest level competed for each group. A Mann–Whitney test indicated that there was no significant difference in age *U* = 1039, *p* = 0.31 or handicap *U* = 671.5, *p* = 0.07. Another factor reported was the athlete’s highest level of competition experienced (school/university, club, county, national, and international). A Mann–Whitney test indicated that there was no significant difference in experience level between the two groups *U* = 2085.5, *p* = 0.07. Finally, the prevalence of choking was 67.7% overall, with specific rates of 75.4 and 61.6% for archery and golf, respectively.

**TABLE 1:** Demographic data for choking and yips groups.

	**Choking Yes**			**Choking No**		
**Characteristic**	***n***	**Mean**	**Standard deviation**	***N***	**Mean**	**Standard deviation**
Age (years)^∗^	*n* = 64; Males = 49; Females = 15	45.41	13.83	*n* = 37; Males = 29; Females = 9	42.49	14.07
Handicap^∗∗^	*n* = 53	8.14	4.89	*n* = 33	10.08	5.28
Experience (Years at highest level)	*n* = 105	12.45	11.36	*n* = 50	9.84	8.84

*Yips type*	*I*	*II*	*III*	*I*	*II*	*III*
Number (%)	*2*	4	38	*6*	2	9
	**Yips Yes**			**Yips No**		
**Characteristic**	***n***	**Mean**	**Standard deviation**	***N***	**Mean**	**Standard deviation**

Age (years)^∗^	*n* = 37; Males = 29; Females = 8	42.41	12.93	*n* = 64; Males = 49; Females = 15	45.45	14.44
Handicap^∗∗^	*n* = 31	8.9	5.28	*n* = 55	8.87	5.05
Experience (Years at highest level)	*n* = 61	10.3	11.32	*n* = *94*	9.29	8.73
*Yips type*	*I*	*II*	*III*	*I*	*II*	*III*
Number (%)	*8*	6	47	Not applicable		

*^∗^not including the 54 participants due to missing data; ^∗∗^just golfers data included. Yips type I = focal-dystonia, II = performace anxiety, III= focal-dystonia and performance anxiety.*

#### Yips

Table 1 provides the mean scores for age, handicap and experience at the highest level competed at for each group. A Mann–Whitney test indicated that there was no significant difference in age *U* = 1022, *p* = 0.25 or handicap *U* = 829, *p* = 0.83. Another factor reported was the athlete’s highest level of competition experience (school/university, club, county, national, and international). A Mann–Whitney test indicated that there was no significant difference in experience level between the two groups *U* = 2750.5, *p* = 0.84. For yips, the prevalence rate was 39.4% overall, with specific rates of 36 and 43.5% for golf and archery, respectively. When reviewing both groups simultaneously, 28.4% of the group experienced both yips and choking (*n* = 44), 11% experienced yips but not choking (*n* = 17), 39.4% experienced choking but not yips (*n* = 61), and the remaining 21.2% experienced neither yips nor choking (*n* = 33).

### Main Analyses Between Groups

A 2 (Choking; Yes, No) × 2 (Yips; Yes, No) × 2 (Sport; Golf, Archery) MANOVA examined main effects and interactions on 20 dependant variables (DV’s; subscales of BFNE, BFI-10, SCS, ASI, PSPS, and FMPS). The results showed that there was a significant multivariate main effect for choking *F*(20,128) = 2.55, *p* = 0.001, Wilk’s λ = 0.76, partial η^2^ = 0.28, and for sport *F*(20,128) = 2.72, *p* < 0.001, Wilk’s λ = 0.70, partial η^2^ = 0.3. There was no significant main effect for yips *F*(20,128) = 1.62, *p* = 0.06, Wilk’s λ = 0.8, partial η^2^ = 0.20. It is worth noting that this missed the significance threshold by a very small margin. There were no significant interactions for choking and yips *F*(20,128) = 0.54, *p* = 0.94, Wilk’s λ = 0.92, partial η^2^ = 0.08; choking and sport *F*(20,128) = 0.87, *p* = 0.62, Wilk’s λ = 0.88, partial η^2^ = 0.12; yips and sport *F*(20,128) = 1.53, *p* = 0.08, Wilk’s λ = 0.81, partial η^2^ = 0.19; and choking, yips, and sport *F*(20,128) = 1.34, *p* = 0.16, Wilk’s λ = 0.83, partial ηη^2^ = 0.17.

#### Choking

Univariate analyses revealed that there was a significant difference between participants who were choking-affected and those who were not on 10 of the 20 variables (see Table 2 which details the means, standard deviation, *F* value and partial η^2^ for each variable). Compared to participants who did not report choking, those who experienced choking reported significantly higher scores for: physical concerns; cognitive concerns; social concerns; FNE; private self-consciousness; non-display of imperfection; concern over mistakes; parental expectations; and doubts about actions and significantly lower levels of conscientiousness.

**TABLE 2:** Total Mean, SD, F value, Partial η^2^ for each variable for both yips and choking groups.

		**Choking**				**Yips**					
		**Yes**	**No**	**Choking**		**Yes**	**No**	**Yips**		**Sport**	
				***F***	**Partial**			***F***	**Partial**	***F***	**Partial**
**Variable**	**Sport**	**Means (SD)**		** value**	**η^2^**	**Means (SD)**		**value**	**η^2^**	**value**	**η^2^**
Fear of negative evaluation	*Archery*	37.44 (12.2)	31.6 (15.09)	10.63^∗∗∗^	0.07	35.17 (13.23)	35.21 (13.14)	0.56	0.004	6.95^∗∗^	0.05
(BFNE-II)	*Golf*	40.58 (12.82)	34 (14.48)			43.35 (12.26)	35.84 (13.5)				
	Total	39.03 (12.56)	33.27 (14.45)			39.32 (13.3)	35.57 (13.28)				
Neuroticism (BFI-10)	*Archery*	2.62 (1.02)	2.29 (0.77)	3.33	0.02	2.72 (0.8)	2.4 (1.07)	2.48	0.02	2.49	0.02
	*Golf*	2.86(1.07)	2.58 (0.90)			3.02 (0.9)	2.6 (1.05)				
	Total	2.74 (1.05)	2.48 (0.86)			2.87 (0.86)	2.52 (1.06)				
Extraversion (BFI-10)	*Archery*	3 (1.3)	2.91 (1.19)	1.27	0.01	3.02 (1.1)	2.95 (1.18)	0.101	0.01	6.81^∗∗^	0.04
	*Golf*	3.24 (0.86)	3.79 (0.97)			3.27 (0.88)	3.54 (0.96)				
	Total	3.11 (1.01)	3.49 (1.12)			3.15 (1)	3.29 (1.09)				
Agreeableness (BFI-10)	*Archery*	3.44 (0.81)	3.38 (0.63)	0.11	0.001	3.37 (0.82)	3.47 (0.73)	0.22	0.001	0.03	0
	*Golf*	3.4 (0.7)	3.48 (0.77)			3.34 (0.64)	3.48 (0.76)				
	Total	3.42 (0.75)	3.45 (0.72)			3.35 (0.73)	3.48 (0.75)				
Conscientiousness (BFI-10)	*Archery*	3.8 (0.9)	4.38 (0.65)	10.74^∗∗∗^	0.07	3.92 (0.98)	3.96 (0.8)	10.74^∗∗∗^	0.07	0.857	0.01
	*Golf*	3.89 (0.86)	4.24 (0.72)			3.53 (0.77)	4.3 (0.72)				
	Total	3.84 (0.88)	4.29 (0.69)			3.72 (0.9)	4.16 (0.77)				
Openness (BFI-10)	*Archery*	3.65 (0.88)	3.56 (1.08)	0.33	0.002	3.37 (0.86)	3.82 (0.86)	1.923	0.01	1.74	0.01
	*Golf*	3.43 (0.84)	3.27 (0.84)			3.32 (0.87)	3.4 (0.83)				
	Total	3.54 (0.87)	3.37 (0.93)			3.34 (0.86)	3.57 (0.9)				
Private Self-Consciousness	*Archery*	3.7 (0.55)	2.62 (0.45)	13.67^∗∗∗^	0.09	2.92 (0.53)	2.98 (0.58)	0.341	0.002	3.32	0.02
(SCS)	*Golf*	3.11 (0.56)	2.84 (0.45)			3.18 (0.62)	2.91 (0.46)				
	Total	3.09 (0.56)	2.77 (0.46)			3.05 (0.58)	2.94 (0.52)				
Public Self-Consciousness	*Archery*	3.08 (0.88)	2.66 (0.91)	1.93	0.02	2.97 (0.9)	2.98 (0.92)	1.192	0.01	13.7^∗∗∗^	0.09
(SCS)	*Golf*	3.43 (78)	3.32 (0.85)			3.65 (0.79)	3.23 (0.78)				
	Total	3.25 (0.84)	3.09 (0.92)			3.32 (0.90)	3.13 (0.84)				
Social Anxiety (SCS)	*Archery*	3.09 (0.56)	2.96 (0.66)	2.19	0.01	3.07 (0.46)	3.05 (0.67)	5.07^∗^	0.03	5.45^∗^	0.04
	*Golf*	3.33 (0.75)	3.06 (0.64)			3.6 (0.65)	3.02 (0.67)				
	Total	3.21 (0.67)	3.03 (0.64)			3.34 (0.62)	3.03 (0.67)				
Physical Concerns (ASI-III)	*Archery*	1.61 (0.75)	1.38 (0.46)	9.39^∗∗^	0.06	1.4 (0.38)	1.68 (0.85)	0.474	0.003	11.76^∗∗∗^	0.07
	*Golf*	2.19 (0.97)	1.7 (0.79)			2.38 (0.12)	1.79 (0.8)				
	Total	1.90 (0.9)	1.59 (0.71)			1.9 (0.92)	1.74 (0.82)				
Cognitive Concerns (ASI-III)	*Archery*	1.62 (0.97)	1.26 (0.35)	12.73^∗∗∗^	0.08	1.41 (0.48)	1.62 (1.06)	2.448	0.016	13.4^∗∗∗^	0.08
	*Golf*	2.24 (0.95)	1.59 (0.68)			2.48 (1.06)	1.71 (0.68)				
	Total	1.93 (1)	1.48 (0.61)			1.96 (0.98)	1.68 (0.86)				
Social Concerns (ASI-III)	*Archery*	2.53 (0.92)	2.28 (0.95)	5.01^∗^	0.03	2.25 (0.87)	2.64 (0.94)	0.09	0.001	2.95	0.02
	*Golf*	2.83 (0.94)	2.4 (85)			2.96 (0.81)	2.5 (0.95)				
	Total	2.68 (0.94)	2.36 (0.88)			2.61 (0.91)	2.56 (0.95)				
Non-Display of Imperfection	*Archery*	3.76 (1.15)	3.28 (1.26)	7.5^∗∗^	0.05	3.82 (1.13)	3.51 (1.23)	6.73^∗∗^	0.04	9.03^∗∗^	0.06
(PSPS)	*Golf*	4.27 (1.08)	3.61 (1.08)			4.66 (1.16)	3.66 (0.93)				
	Total	4.02 (1.14)	3.5 (1.14)			4.25 (1.21)	3.6 (1.06)				
Non-Disclosure of Imperfection	*Archery*	4.06 (0.79)	3.77 (0.78)	1.24	0.01	3.94 (0.87)	4.02 (0.74)	3.353	0.02	9.45^∗∗^	0.06
(PSPS)	*Golf*	4.25 (0.77)	4.16 (0.72)			4.63 (0.76)	3.98 (0.63)				
	Total	4.16 (0.78)	4.03 (0.75)			4.29 (0.88)	4 (0.67)				
Perfectionistic Self-Promotion	*Archery*	3.94 (1.02)	3.68 (0.92)	2.429	0.02	3.97 (0.92)	3.8 (1.05)	6.44^∗^	0.04	5.1^∗^	0.03
(PSPs)	*Golf*	4.2 (1.01)	3.91 (0.86)			4.67 (0.9)	3.76 (0.84)				
	Total	4.07 (1.02)	3.83 (0.88)			4.32 (0.97)	3.78 (0.93)				
Concern Over Mistakes	*Archery*	2.47 (1.11)	1.89 (1.02)	10.57^∗∗∗^	0.07	2.44 (1.1)	2.24 (1.12)	2.23	0.02	1.27	0.01
(FMPS)	*Golf*	2.53 (0.87)	2.04 (0.73)			2.73 (0.79)	2.11 (0.8)				
	Total	2.48 (0.99)	1.99 (0.83)			2.59 (0.96)	2.17 (0.94)				
Organisation (FMPS)	*Archery*	3.26 (0.88)	3.44 (0.72)	1.61	0.01	3.3 (0.91)	3.31 (0.8)	2.1	0.14	3.5	0.02
	*Golf*	3.59 (0.85)	3.59 (0.85)			3.38 (0.78)	3.88 (0.84)				
	Total	3.43 (0.88)	3.43 (0.88)			3.34 (0.84)	3.65 (0.87)				
Personal Standards (FMPS)	*Archery*	3.68 (0.84)	3.71 (0.66)	0.12	0.001	3.7 (0.75)	3.67 (0.83)	0	0	0.07	0
	*Golf*	3.63 (0.81)	3.65 (0.65)			3.6 (0.73)	3.65 (0.77)				
	Total	3.65 (0.82)	3.67 (0.65)			3.65 (0.74)	3.66 (0.79)				
Parental Expectations (FMPS)	*Archery*	2.32 (1.03)	1.86 (0.85)	9.9^∗∗^	0.06	2.17 (0.99)	2.23 (1.02)	2.71	0.02	0.2	0.001
	*Golf*	2.38 (0.91)	1.76 (0.84)			2.59 (0.98)	1.89 (0.8)				
	Total	2.35 (0.96)	1.8 (0.83)			2.39 (1)	2.03 (0.91)				
Doubts About Action (FMPS)	*Archery*	2.78 (0.58)	2.25 (0.88)	6.57^∗^	0.04	2.63 (0.69)	2.5 (0.96)	2.96	0.02	1.74	0.01
	*Golf*	3.08 (0.77)	2.35 (1.02)			2.98 (0.85)	2.44 (0.90)				
	Total	2.92 (0.69)	2.32 (0.96)			2.81 (0.79)	2.46 (0.92)				

*^∗^Significant at the 0.05 level; ^∗∗^Significant at the 0.01 level; ^∗∗∗^Significant at *p* < 0.001 level.*

#### Yips

Univariate analyses revealed significant effects for four of the 20 variables between those who were yips-affected and those who were not (see Table 2; which details the means, standard deviation, *F* value and partial η^2^ for each variable). Participants who experienced the yips reported significantly higher social anxiety, non-display of imperfection, and perfectionistic self-promotion and significantly lower scores for conscientiousness, compared with those who did not experience the yips.

#### Sport

Univariate analyses revealed that there were differences between golfers and archers on nine of the 20 variables (see Table 2 which details the means, standard deviation, *F* value and partial η^2^ for each variable). Golfers reported significantly higher scores than archers for: physical concerns; cognitive concerns; FNE; extraversion; public self-consciousness; social anxiety; non-display of imperfection; non-disclosure of imperfection; and perfectionistic self-promotion.

### Analyses of Two Predictive Models

#### Choking

A discriminant function analysis was conducted to test if the significant variables revealed in the MANOVA could act as predictors for whether an individual has experienced a choke or not. This predictive model included the 10 variables that differed in univariate analyses: physical concerns, cognitive concerns, social concerns, FNE, conscientiousness, private self-consciousness, non-display of imperfection, concern over mistakes, parental expectations, and doubts about actions, which revealed one discriminant function, canonical *R*^2^ = 0.41, and significantly differentiated the groups, λ = 0.83, *X*^2^_(__2__)_ = 27.32, *p* = 0.002 (see Table 3 for full detail on how the model was loaded). Conscientiousness (negative) and private self-consciousness were the largest contributors to the model. This model was able to predict 71% of the original sample successfully into correct groups.

**TABLE 3:** The Standardized canonical discriminant function coefficients and the correlations between the observed variables for choking.

	**Standardized canonical**	**Structure**
	**discriminant**	**matrix**
	**function coefficient**	
	
	**Function**	
Characteristic		
Physical concerns	–0.17	0.38
Cognitive concerns	0.25	0.54
Social concerns	–0.21	0.37
Fear of negative evaluation	0.2	0.49
Conscientiousness	–0.57	–0.57
Private self-consciousness	0.51	0.63
Non-display of imperfection	–0.13	0.48
Concern over mistakes	0.29	0.57
Parental expectations	0.39	0.62
Doubts about actions	–0.16	0.5
		

#### Yips

A discriminant function analysis was conducted to test if the significant variables revealed in the MANOVA could act as predictors for whether an individual has experienced the yips or not. This predictive model included the four variables reported in section two: conscientiousness, social anxiety, non-display of imperfection and perfectionistic self-promotion, which revealed one discriminant function, canonical *R*^2^ = 0.37, and significantly differentiated the groups, λ = 0.87, *X*^2^_(__2__)_ = 21.57, *p* = 0.002 (see Table 4 for full detail on how the model was loaded). Conscientiousness and perfectionistic self-promotion were the largest contributors to the model. This model was able to predict 69% of the original sample successfully into correct groups.

**TABLE 4:** The Standardized canonical discriminant function coefficients and the correlations between the observed variables for yips.

	**Standardized canonical**	**Structure**
	**discriminant**	**matrix**
	**function coefficient**	
	
	**Function**	
Characteristic		
Conscientiousness	–0.59	0.73
Social Anxiety	0.39	0.73
Non-display of imperfection	–0.01	–0.67
Perfectionistic self-promotion	0.52	0.59

## Discussion

**FIGURE 4:**
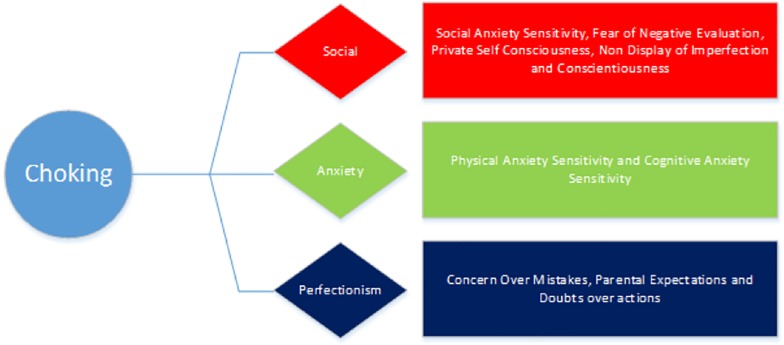
The Choking Predictive Model (CPM).

**FIGURE 5:**
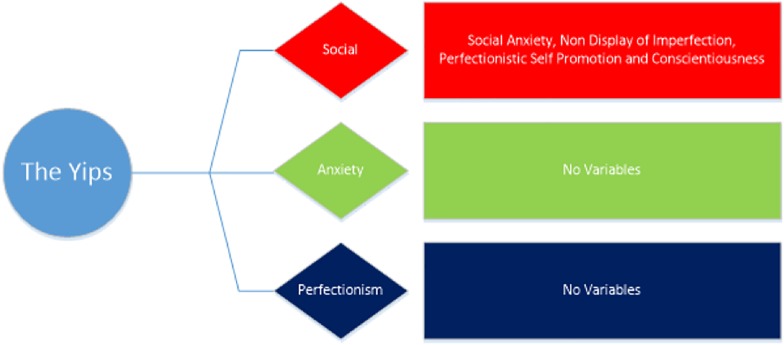
The Yips Predictive Model (YPM).

The role of personality traits in predicting susceptibility to experience paradoxical performances has emerged in recent reports (Bennett et al., 2016; Laborde et al., 2019), yet this research is still in its infancy. As such, the primary aim of the current study was to investigate whether individual differences could predict the prevalence of choking and/or the yips in a large sample of competitive golfers and archers. It was hypothesized that several social, anxiety and perfectionism variables would be significantly higher in those who experienced choking and the yips compared to non-affected athletes (see Figures 2, 3). A supplementary MANOVA revealed no significant interaction for choking and the yips collectively, suggesting that the predictors for each sub group (choking and yips) were independent. Within the choking group, there was partial support for the hypothesis as four social variables, two anxiety variables, and three perfectionism variables were significantly higher, and one social variable (conscientiousness) was significantly lower, in those choking-affected athletes compared to those unaffected. A discriminant function analysis revealed that together these 10 variables predicted 71% of the original sample correctly, with conscientiousness and private self-consciousness as the largest contributors to the Choking Predictive Model (CPM: see Figure 4). In contrast, within the yips model, only social variables were significantly different with perfectionistic self-promotion, social anxiety and non-display of imperfection being significantly higher and conscientiousness being significantly lower in those yips-affected athletes compared to their unaffected counterparts; these findings partially supported the hypothesis. Discriminant function analysis revealed that these four variables predicted 69% of the original sample correctly, with conscientiousness and perfectionistic self-promotion as the largest contributors to the Yips Predictive Model (YPM: see Figure 5). It is noteworthy that only two predictors were consistent across both models in conscientiousness and non-display of imperfection, further highlighting that that the predictors for each sub group were independent. This is the first study, to the authors knowledge, that investigates paradoxical performances using a range of anxiety, social, and perfectionism variables collectively with a large sample (*n* = 155) of experienced competitive athletes (Geukes et al., 2012; Mesagno et al., 2012; Roberts et al., 2013). Moreover, this highlights the significantly different personality patterns associated with both choking (combination of social, anxiety, and perfectionism) and yips (social) susceptibility in archers and golfers. The implications of anxiety, social, and perfectionism factors will be explored in this section.

The current study revealed a prevalence rate of 39.4% for athletes who have experienced yips in golf and archery. This number is consistent with previous research which has suggested prevalence rates of between 16 and 54% (McDaniel’s et al., 1989; Smith et al., 2003; Klämpfl et al., 2013a). Although the current rates are consistent with previous literature, the vast difference across studies outlines the potential reliance on researchers to focus on self-report as a mean of identifying yips affected versus using more comprehensive measurements of assessing the prevalence of yips, such as kinematic measurements. However, it is worth noting that the aim of the current study was not to explore traits in those who are currently experiencing the yips, but in understanding predictors of yips experience. Within the sample, the prevalence of choking was recorded as 67.7%. This is the first study to the authors’ knowledge to report the prevalence rate for athletes who have experienced choking and showcase the importance of understanding choking and performance under pressure given the high prevalence rate. Interestingly, this study is the first to investigate choking and yips simultaneously reporting that of the 39.4% of athletes that had experienced the yips, 28.4% of those athletes had experienced choking. This provides some evidence that there may be some similarities between the experience of choking and the yips (e.g., Bennett et al., 2015), but that there are also differences as 11% experienced yips only without ever experiencing choking. This provides support for those reviews suggesting that the yips and choking are completely different forms of performance breakdown (Hill et al., 2010). For instance, predictors of both the yips and choking stemmed from social origins, but the specific traits were different in both. As the current study was based on subjective responses to having experienced paradoxical performance, further experimental testing of these paradoxical experiences is warranted in laboratory or ecologically valid (competition) settings under different social manipulations to see their role in yips and choking susceptibility (Lobinger et al., 2014; Clarke et al., 2015).

When reviewing the role of anxiety factors within both YPM and CPM, the current findings illustrate that higher levels of anxiety sensitivity originating from physical, cognitive and social sources are exhibited in choking-affected athletes but not within yips-affected athletes. This is the first study to investigate anxiety interpretation using a trait measure of sensitivity. It is well documented that anxiety is a consistent predictor of choking, yet its exact role is unclear (Hill et al., 2010). Research has already suggested that high levels of trait anxiety can induce a choking experience (Wilson, 2008), yet this is not to say that individuals with low levels of trait anxiety will not experience a choke. The current findings support qualitative accounts of choking (Guicciardi et al., 2010), which suggest that athletes’ sensitivity to changes in bodily cues, such as cognitive and somatic arousal, may provide greater insight into the anxiety-performance relationship than intensity alone. As such, Schmidt et al. (1997) suggested that if individuals interpret changes in bodily cues due to an increase in arousal in a fearful manner, are likely to exhibit increases in anxiety and apprehension. Anxiety sensitivity refers to the fear of anxiety related sensations and the associated negative consequences (Deacon et al., 2003). Of interest, the Directional Interpretation Hypothesis (Jones and Hanton, 2001) identifies that interpretation of anxiety symptoms may be more important than intensity of anxiety symptoms on performance, particularly cognitive anxiety interpretation (Butt et al., 2003). Thus, individuals who experience higher fear of anxiety-related sensations are more likely to interpret arousal negatively. This is of interest, as ACT (Eysenck and Derakshan, 2011) suggests that if the finite attentional resources are consumed by irrelevant cues (e.g., cognitive anxiety), a deterioration in performance is likely to occur as athletes do not address key performance cues. As such, future choking research should further investigate the influence of both anxiety interpretation and sensitivity on specific biomechanical and psycho-physiological parameters of performance (Cooke et al., 2010).

Interestingly, when exploring levels of neuroticism, there were no differences between those who experienced either paradoxical performance and those who did not. This was unexpected as previous research has suggested a positive association between anxiety and neuroticism (Muris et al., 2005) and that choking occurs under high levels of anxiety and pressure (Guicciardi et al., 2010). In addition, Byrne et al. (2015) reported higher levels of neuroticism as a key predictor of poor performance under pressure in decision-making tasks. When reviewing the other factors from the Big-Five Personality Model (McCrae and Costa, 2008) conscientiousness was a significant predictor of both choking and yips and social anxiety for just yips. Conscientiousness refers to when individuals are goal-directed, delay gratification and follows norms and rules (Roberts et al., 2009). This was the largest contributor and negative predictor within both the CPM and the YPM, which suggests that those individuals who attempt to refrain from acting within social norms, are less conscientious, less careful and more likely to take risks, are more likely to experience the yips and choking. This is the first time the Big Five personality factors have been investigated in relation to the yips. To date, only Byrne et al.’s (2015) multi-study paper has explored the Big Five Factors in pressure performances and the findings were inconclusive. However, Woodman et al. (2010) revealed that conscientiousness was positively associated with an athlete’s quality of preparation in the lead up to competition, suggesting higher levels of conscientiousness are related to greater competition preparation. This may indicate that individuals are more likely to choke or experience a yip when they do not effectively prepare for competition. Yet caution is warranted when interpreting the current findings, as there were issues with reliability with the BFI-10 (Rammstedt and John, 2007) and the measure is a reduced item scale, with only two items per factor (Chen et al., 2001). Therefore, the role of neuroticism may still play a key role in understanding those who are susceptible to experience both forms paradoxical performance, yet a more reliable and robust measure of this should be utilized. As such, further investigation using the BFI (John et al., 1991) may provide greater insight into the role of conscientiousness and neuroticism within both yips and choking experience.

Next, when considering the role of factors stemming from social sources, within the YPM, social anxiety, perfectionistic self-promotion, and non-display of imperfection were all significantly higher in those yips-affected athletes compared to those non-affected highlighting the important role social factors have in the yips experience. This is not surprising given the physical symptoms associated with the yips can often be visible to observers (competitors, fans etc.). In comparison, symptoms experienced by an individual experiencing a choke may manifest in symptoms that are not always visible to observers, such as cognitive anxiety or FNE. The strongest predictor of these factors was perfectionistic self-promotion, whereby yips-affected athletes attempt to project an image of fitting in perfectly with a social situation (Flett and Hewitt, 2014). Furthermore, the high levels of non-display of imperfection observed suggests that yips-affected athletes defensively cover up mistakes more than their unaffected counterparts. Flett and Hewitt (2014) proposed an expanded model of perfectionism and social anxiety suggesting that perfectionism factors such as perfectionistic self-presentation, perfectionistic rumination/mistake rumination and perfectionistic discrepancies act as a predictor of social anxiety. Hewitt et al. (2003) suggest that high levels of perfectionistic self-promotion, in combination with a desire to cover imperfections, may originate from a compensatory mechanism used to protect against a low or fragile sense of self-acceptance, and a sense of not belonging or not being accepted by others. Perfectionistic self-promotion and non-display of imperfection have been linked to social anxiety in several studies (Hewitt et al., 2003; Nepon et al., 2011). Furthermore, perfectionistic self-promotion, non-display of imperfection and non-disclosure of imperfection are robust predictors of daily social anxiety (Mackinnon et al., 2014). Although non-disclosure of imperfection was not included in the current YPM, it was approaching significance within the analysis, and it was a factor within the CPM. Flett et al. (2014) also reported that those who experience higher levels of perfectionistic self-promotion experience a high need for validation: for example, a need to prove their sense of worth. Non-display of imperfection was also identified as a robust predictor of cluster C traits, which is the anxious and fearful cluster of the DSM-5 American Psychiatric Association [APA], 2013; Sherry et al., 2007). Furthermore, these self-presentational perfectionism concerns have also been linked with frequent intrusive automatic thoughts about the need to be perfect which increase social anxiety (Sturman, 2011), anxiety sensitivity (Flett et al., 2004), and insecure attachment style (Boone, 2013). Interestingly, the current study found no difference between both yips-affected and non-affected groups for anxiety sensitivity, it should be noted that both groups exhibited higher levels of social concerns compared to cognitive and somatic concerns. Furthermore, this is the first study to investigate the role of perfectionistic self-presentation within a sporting sample, and as such no comparisons can be made with other sporting literature on this topic. As such, further research on each of the perfectionistic self-presentation traits and their role within paradoxical performance experience is warranted.

Within the CPM, the findings revealed that social anxiety concerns, FNE, private self-consciousness, and non-display of imperfection were higher in those choking-affected athletes, highlighting the role that factors related to self-consciousness play within the choking experience. These findings support experimental evidence that higher private self-consciousness (self-focus), but not public self-consciousness was reported in those who experienced choking (Geukes et al., 2012). This proposal was partially supported within the current sample as there were no differences in public self-consciousness (distraction) between those who were choking-affected and those who were unaffected, suggesting that individuals who choke tend to internalize their focus.

Other sources of distraction self-consciousness, in the form of FNE and non-display of imperfection, were significantly higher in athletes who reported choking compared to those who did it. These findings support Mesagno et al. (2011) proposal that self-presentational concerns may be a potential origin for choking. Furthermore, Leary (1992) suggests that competitive anxiety revolves around self-presentational implications of competition. Both constructs involve athletes not wanting to be negatively evaluated by others (Hewitt et al., 2003; Mesagno et al., 2012). Therefore, it is possible that both self-focus and distraction forms of self-consciousness are integral components to the anxiety-performance relationship. This is particularly important as private self-consciousness could be explained by self-focus models of choking (Masters, 1992) as athletes focus their attention inward to controlling movement. Whereas, social forms of self-consciousness could be explained by distraction models of choking (Eysenck et al., 2007); athletes fail to focus on key performance cues when they are distracted by irrelevant cues. This would support the assumption highlighted by Mesagno and Marchant (2013) who identified that self-focus and distraction models of choking could be investigated separately, whereby individuals high in trait measures of private self-consciousness would increase levels of self-focus during pressure environments. In addition, those who experience high trait levels of social self-consciousness may be predisposed to increase public self-awareness when experiencing pressure and focus their attention on avoiding negative judgment or perceptions from the audience. Future research investigating these characteristics as an explanation for both self-focus and distraction models of choking is needed in studies that create different pressure environments.

Finally, factors that stemmed from perfectionism sources had different influence on both the CPM and the YPM. Within the proposed CPM, athletes with higher levels of three perfectionism measures (concern over mistakes, parental expectation, doubts over actions) were more likely to experience choking. Research suggests that the subcomponents of Frost et al.’s (1990) model of perfectionism can be divided into two broad dimensions; (i) perfectionistic strivings, which includes individuals setting high personal standards and striving for perfection, and (ii) perfectionistic concerns which involves individuals being highly critical in self-evaluation (e.g., Dunkley et al., 2003; Stoeber and Otto, 2006). Furthermore, healthy perfectionists exhibit high levels of perfectionistic strivings and low levels of perfectionistic concerns, whereas unhealthy perfectionists display high levels of both perfectionistic concerns and strivings (Stoeber and Otto, 2006). Choking-affected athletes in the current study had a less healthy perfectionism profile than those who were not affected. Collectively, these findings support the previous proposal that unhealthy perfectionists experience higher levels of FNE, anxiety, and anxiety sensitivity than healthy perfectionists (Kawamura et al., 2001; Koivula et al., 2002).

The proposed YPM suggested that there were no differences in perfectionism between both affected and non-affected athletes unlike that witnessed in previous yips research (Roberts et al., 2013; Bennett et al., 2016). Interestingly, the means observed in the current study for trait multidimensional perfectionism (Frost et al., 1990) variables were indeed higher than those reported in the Roberts et al. (2013) study but were not significantly different to those non-affected athletes. Indeed Roberts et al. recognize that the mean scores for doubts about actions, personal standards, organization, and concern over mistakes for those yips-affected athletes were low compared to other studies investigating perfectionism, and this may suggest why the current study found no significant differences. These findings do support the findings of Klämpfl et al. (2013b) that there were no differences between those yips-affected and those not. However, we support Roberts et al.’s suggestion that future research should incorporate a sport specific measure of trait perfectionism to provide key insight into the role this plays in the experience of the yips.

When considering our demographic data, the findings revealed that there was no significant difference in age within the two groups within both forms of paradoxical performances (Choking: yes/no; Yips: yes/no). This is of interest for those experiencing the yips as it provides support to previous research that has identified no difference in age between those yips-affected and non-affected (see Clarke et al., 2015 for a full review). To date, only two yips studies have reported a difference between yips-affected and unaffected golfers; Adler et al. (2011) and Stinear et al. (2006) reported that yips-affected athletes were significantly older than those unaffected athletes, suggesting that experience may be a pathway for yips development, and specifically that overuse of motor skills may act as one possible mechanism (Smith et al., 2003). However, analysis of the demographics also revealed no significant difference in experience or handicap, in golfers, between the two groups within each paradoxical performance, supporting that individuals of all levels and experience can suffer with these symptoms (Clarke et al., 2015). Only golfers’ current handicaps were recorded which may not be the best indicator of ability as Adler et al. (2011) reported that those golfers who reported experiencing the yips had a significantly lower best handicap than those non-affected. This may be a better indicator of the impact of the yips as the onset of the yips may contribute to an increase in handicap.

The current findings suggest some practical implications worth highlighting. First, the current study has provided several potential predictors for those likely to experience a yip or choking experience. This study has also shown the complexity of choking and the yips, given the range of different psychological traits that play a role in each and previous qualitative accounts of each (Guicciardi et al., 2010; Bennett et al., 2016). This suggests that the experience of choking or the yips may include a range of factors that practitioners and coaches need to be aware of and consider when understanding their athletes experience of the yips or choking. As such, the CPM and YPM provides a model of factors to help inform practitioners and coaches on those athletes who are more susceptible to experiencing these paradoxical performances (Hill et al., 2010; Lobinger et al., 2014). For instance, individuals who report higher levels of anxiety sensitivity, self-presentational concerns or perfectionism are likely to experience choking and yips behavior. As such, practitioners can develop tailored interventions to help clients cope more effectively with pressured environments, to ensure they remain in a consistent, positive and confident mind-set for performance (Clarke et al., 2015; Mine et al., 2018). Furthermore, coaches can create environments for athletes to test these strategies in the safety of a training environment. Specifically, as these findings encourage coaches to refrain from using social comparison in their communication to athletes, given the increased influence of self-consciousness in both the yips and choking.

A potential limitation of the current study was that the classification of both yips and choking was based on self-report. This is particularly pertinent within the yip’s literature as recent research by Klämpfl et al. (2013a) suggested that future research should use more objective yips criterion like screening tests to classify athletes. As the current study was investigating psychological traits of individuals with the yips, the use of self-report was considered the most effective and appropriate approach to access a large sample of participants. However, we support the suggestion that when conducting laboratory studies, a more objective criterion is warranted particularly when investigating the different mechanisms during high-pressure environments. However, some of the limitations of exploring yips in laboratory studies is that individuals who identify as being yips affected may not experience observable symptoms and lack ecological validity (Smith et al., 2000). Furthermore, it is acknowledged that given the cross-sectional design utilized in the current study, conclusions about causality of both forms of paradoxical performance cannot be drawn, but the findings highlight these predictors increase the susceptibility of athletes to experience it. Consequently, it is not possible to ascertain whether these psychological traits are psycho-reactive or pre-existent to the yips or choking experience. Therefore, future research needs to adopt both longitudinal and intervention-based research aimed at specific traits to better understand these factors as potential causes or consequences of the yips and choking.

## Conclusion

In conclusion, this study addressed Lobinger et al.’s (2014) call for research investigating psychological characteristics as potential correlates of paradoxical performances. The current study is the first to explore the role of perfectionism self-presentation within sport and is the first study to investigate a wide range of psychological traits in the experience of the yips and choking. Our findings emphasize the role personality traits play in the susceptibility of paradoxical performances, particularly the role of perfectionistic self-presentation in experiencing both yips and choking. The current study also provides a novel approach by investigating two of the most popular paradoxical performances in yips and choking simultaneously. Accordingly, we propose two predictive models of paradoxical performance: the yips model comprising social factors solely, where the choking model includes social, anxiety and perfectionism factors.

## Data Availability Statement

The datasets generated for this study are available on request to the corresponding author.

## Ethics Statement

The studies involving human participants were reviewed and approved by the University of Derby Human Sciences. The patients/participants provided their written informed consent to participate in this study.

## Author Contributions

All authors contributed to the conception and design of the study, wrote sections of the manuscript, and manuscript revision, read and approved the submitted version. PC organized the database and wrote the first draft of the manuscript. DS performed the statistical analysis.

## Conflict of Interest

The authors declare that the research was conducted in the absence of any commercial or financial relationships that could be construed as a potential conflict of interest.

## References

[B1] Adler C. H. Crews D. Kahol K. Santello M. Noble B. Hentz J. G. (2011). Are the yips a task-specific dystonia or “golfer’s cramp?” *Mov. Disord.* 26 1993–1996. 10.1002/mds.23824 21674625

[B2] Allen M. S. Greenless I. Jones M. (2013). Personality in sport: a comprehensive review. *Int. Rev. Sport Exerc. Psychol.* 6 184–208. 10.1080/1750984x.2013.769614

[B3] Altenmüller E. Jabusch H. C. (2009). Focal hand dystonia in musicians: phenomenology, etiology and psychological trigger factors. *J. Hand Ther.* 22 144–155. 10.1016/j.jht.2008.11.007 19278826

[B4] American Psychiatric Association [APA] (2013). *Diagnostic and Statistical Manual of Mental Disorders (DSM-5^®^).* Maine Avenue: American Psychiatric Association.

[B5] Baumeister R. F. (1984). Choking under pressure: self-consciousness and paradoxical effects of incentives on skilful performance. *J. Pers. Soc. Psychol.* 46 610–620. 10.1037//0022-3514.46.3.610 6707866

[B6] Baumeister R. F. Showers C. J. (1986). A review of paradoxical performance effects: choking under pressure in sports and mental tests. *Eur. J. Soc. Psychol.* 16 361–383. 10.1002/ejsp.2420160405

[B7] Bawden M. Maynard I. (2001). Towards an understanding of the personal experience of the “yips” in cricketers. *J. Sport Sci.* 19 937–953. 10.1080/026404101317108444 11820688

[B8] Bell S. T. (2007). Deep-level composition variables as predictors of team performance: a meta-analysis. *J. Appl. Psychol.* 92:595. 10.1037/0021-9010.92.3.595 17484544

[B9] Bennett J. Hays K. Lindsay P. Olusoga P. Maynard I. W. (2015). Yips and lost move syndrome: exploring psychological symptoms, similarities and implications for treatment. *Int. J. Sport Psychol.* 46 61–82.

[B10] Bennett J. Rotherham M. Hays K. Olusoga P. Maynard I. W. Lindsay P. (2016). Yips and lost move syndrome: assessing impact and exploring levels of perfectionism, rumination and reinvestment. *Sport Exerc. Psychol. Rev.* 12 14–27.

[B11] Besser A. Flett G. L. Hewitt P. L. (2010). Perfectionistic self-presentation and trait perfectionism in social problem-solving ability and depressive symptoms. *J. Appl. Soc. Psychol.* 40 2121–2154. 10.1111/j.1559-1816.2010.00653.x

[B12] Boone L. (2013). Are attachment styles differentially related to interpersonal perfectionism and binge eating? *Pers. Individ. Dif.* 54 931–935. 10.1016/j.paid.2013.01.006

[B13] BPS (2013). *Report of the Working Party on Conducting Research on the Internet: Ethics Guidelines for Internet-Mediated Research.* Leicester: British Psychological Society.

[B14] Butt J. Weinberg R. Horn T. (2003). The intensity and directional interpretation of anxiety: fluctuations throughout competition and relationship to performance. *Sport Psychol.* 17 35–54. 10.1123/tsp.17.1.35

[B15] Byrne K. A. Silasi-Mansat C. D. Worthy D. A. (2015). Who chokes under pressure? The big five personality traits and decision-making under pressure. *Pers. Individ. Diff.* 74 22–28. 10.1016/j.paid.2014.10.009 28373740 PMC5376094

[B16] Carleton R. N. McCreary D. R. Norton P. J. Asmundson G. J. (2006). Brief fear of negative evaluation scale–revised. *Depress. Anxi.* 23 297–303. 10.1002/da.20142 16688736

[B17] Chen F. Bollen K. A. Paxton P. Curran P. J. Kirby J. B. (2001). Improper solutions in structural equation models causes, consequences, and strategies. *Sociol. Methods Res.* 29 468–508. 10.1177/0049124101029004003

[B18] Clark T. P. Tofler I. R. Lardon M. T. (2005). The sport psychiatrist and golf. *Clin. Sports Med.* 24 959–971. 10.1016/j.csm.2005.04.001 16169456

[B19] Clarke P. Sheffield D. Akehurst S. (2015). The yips in sport: a systematic review. *Int. Rev. Sport Exerc. Psychol.* 8 156–184. 10.1080/1750984x.2015.1052088

[B20] Cooke A. Kavussanu M. McIntyre D. Ring C. (2010). Psychological, muscular and kinematic factors mediate performance under pressure. *Psychophysiology* 47 1109–1118. 10.1111/j.1469-8986.2010.01021.x 20409012

[B21] Deacon B. J. Abramowitz J. S. Woods C. M. Tolin D. F. (2003). The anxiety sensitivity index-revised: psychometric properties and factor structure in two nonclinical samples. *Behav. Res. Ther.* 41 1427–1449. 10.1016/s0005-7967(03)00065-2 14583412

[B22] Dunkley D. M. Zuroff D. C. Blankstein K. R. (2003). Self-critical perfectionism and daily affect: dispositional and situational influences on stress and coping. *J. Pers. Soc. Psychol.* 84 234–252. 10.1037/0022-3514.84.1.234 12518982

[B23] Eysenck M. W. Derakshan N. (2011). New perspectives in attentional control theory. *Pers. Individ. Diff.* 50 955–960. 10.1111/j.1467-8624.2012.01738.x 22364371

[B24] Eysenck M. W. Derakshan N. Santos R. Calvo M. G. (2007). Anxiety and cognitive performance: attentional control theory. *Emotion* 7 336–353. 10.1037/1528-3542.7.2.336 17516812

[B25] Fenignstein A. Scheierm M. F. Buss A. H. (1975). Public and private self-consciousness: assessment and theory. *J. Consult. Clin. Psychol.* 43 522–527. 10.1037/h0076760

[B26] Field A. (2013). *DISCOVERING Statistics Using IBM SPSS Statistics.* Thousand Oaks, CA: Sage.

[B27] Flett G. L. Besser A. Hewitt P. L. (2014). Perfectionism and interpersonal orientations in depression: an analysis of validation seeking and rejection sensitivity in a community sample of young adults. *Psychiatr. Interpers. Biol. Process.* 77 67–85. 10.1521/psyc.2014.77.1.67 24575914

[B28] Flett G. L. Grenne A. Hewitt P. L. (2004). Dimensions of perfectionism and anxiety sensitivity. *J. Ration. Emot. Cogn. Behav. Ther.* 22 37–55.

[B29] Flett G. L. Hewitt P. L. (2014). The perils of perfectionism in sports” revisited: toward a broader understanding of the pressure to be perfect and its impact on athletes and dancers. *Int. J. Sport Psychol.* 45 395–407.

[B30] Frost R. O. Marten P. Lahart C. Rosenblate R. (1990). The Dimensions of perfectionism. *Cogn. Ther. Res.* 14 449–468.

[B31] George D. Mallery P. (2003). *SPSS for Windows Step by Step: A Simple Guide and Reference. 11.0 Update*, 4th Edn, Boston: Allyn & Bacon.

[B32] Geukes K. Mesagno C. Hanrahan S. Kellman M. (2012). Testing an interactionist perspective on the relationship between personality traits and performance under public pressure. *Psychol. Sport Exerc.* 13 243–250. 10.1016/j.psychsport.2011.12.004

[B33] Guicciardi D. F. Longbottom J. L. Jackson B. Dimmock J. A. (2010). Experienced golfers perspectives on choking under pressure. *J. Sport Exerc. Psychol.* 32 61–83. 10.1123/jsep.32.1.61 20167952

[B34] Hewitt P. L. Flett G. L. Sherry S. B. Habke M. Parkin M. Lam R. W. (2003). The interpersonal expression of perfection: perfectionistic self-presentation and psychological distress. *J. Pers. Soc. Psychol.* 84:1303. 10.1037/0022-3514.84.6.1303 12793591

[B35] Higgins E. T. (1998). Promotion and prevention: regulatory focus as a motivational principle. *Adv. Exp. Soc. Psychol.* 30 1–46. 10.1016/s0065-2601(08)60381-0

[B36] Hill D. M. Hanton S. Matthews N. Fleming S. (2010). Choking in sport: a review. *Int. Rev. Sport Exerc. Psychol.* 3 24–39. 10.1080/17509840903301199

[B37] Hobden K. Pliner P. (1995). Self-handicapping and dimensions of perfectionism: Self-presentation vs self-protection. *J. Res. Pers.* 29 461–474. 10.1006/jrpe.1995.1027

[B38] Ioannou C. Klämpfl M. Lobinger B. Raab M. Altenmüller E. (2018). Psychodiagnostics: classification of the yips phenomenon based on musician’s dystonia. *Med. Sci. Sports Exerc.* 50 2217–2225. 10.1249/mss.0000000000001696 29933350

[B39] John O. P. Donahue E. M. Kentle R. L. (1991). *The Big Five Inventory—Versions 4a and 54.* Berkeley, CA: University of California.

[B40] Jones G. Hanton S. (2001). Pre-competitive feeling states and directional anxiety interpretations. *J. Sports Sci.* 19 385–395. 10.1080/026404101300149348 11411775

[B41] Kawamura K. Y. Hunt S. L. Frost R. O. DiBartolo P. M. (2001). Perfectionism, anxiety, and depression: are the relationships independent? *Cogn. Ther. Res.* 25 291–301. 25543609

[B42] Klämpfl M. K. Lobinger B. H. Raab M. (2013a). Reinvestment-The cause of the yips? *PLoS One* 8:e82470. 10.1371/journal.pone.0082470 24340032 PMC3855447

[B43] Klämpfl M. K. Lobinger B. H. Raab M. (2013b). How to detect the yips in golf. *Hum. Mov. Sci.* 32 1270–1287. 10.1016/j.humov.2013.04.004 24016710

[B44] Koivula N. Hassmen P. Fallby J. (2002). Self-esteem and perfectionism in elite athletes: Effects on competitive anxiety and self-confidence. *Pers. Individ. Diff.* 32 865–875. 10.1016/s0191-8869(01)00092-7

[B45] Laborde S. Allen M. S. Katschak K. Mattonet K. Lachner N. (2019). Trait personality in sport and exercise psychology: a mapping review and research agenda. *Int. J. Sport Exerc. Psychol.* 23 1–16. 10.1080/1612197x.2019.1570536

[B46] Leary M. R. (1992). Self-presentational processes in exercise and sport. *J. Sport Exerc. Psychol.* 14 339–339.

[B47] Leary M. R. (2001). “Shyness and the self: attentional, motivational, and cognitive self-processes in social anxiety and inhibition,” in *International Handbook of Social Anxiety: Concepts, Research and Interventions Relating to the Self and Shyness*, eds Crozier W. R. Alden L. E. (New York, NY: John Wiley & Sons Ltd.), 217–234.

[B48] Lehn A. Mellick G. Boyle R. (2014). Psychiatric disorders in idiopathic-isolated focal dystonia. *J. Neurol.* 261 668–674. 10.1007/s00415-014-7244-8 24449065

[B49] Lencer R. Steinlechner S. Stahlberg J. Rehling H. Orth M. Baeumer T. (2009). Primary focal dystonia: evidence fo distinct neuro-psychiatric and personality profiles. *J. Neurol. Neurosurg. Psychiatr.* 80 1176–1179. 10.1136/jnnp.2008.170191 19465414

[B50] Lobinger B. H. Klämpfl M. K. Altenmuller E. (2014). We are able, we intend, we act - but we do not succeed: a theoretical framework for a better understanding of paradoxical performance in sports. *J. Clin. Sport Psychol.* 8 357–377. 10.1123/jcsp.2014-0047

[B51] Mackinnon S. P. Battista S. R. Sherry S. B. Stewart S. H. (2014). Perfectionistic self-presentation predicts social anxiety using daily diary methods. *Pers. Individ. Diff.* 56 143–148. 10.1016/j.paid.2013.08.038

[B52] Masters R. S. (1992). Knowledge, knerves and know-how: the role of explicit versus knowledge in the breakdown of complex motor skills under pressure. *Br. J. Psychol.* 83 343–385. 17470398

[B53] McCrae R. R. Costa P. T. (2008). Empirical and theoretical status of the five-factor model of personality traits. *SAGE Handb. Pers. Theory Assess.* 1 273–294. 10.4135/9781849200462.n13

[B54] McDaniel’s K. D. Cummings J. L. Shain S. (1989). The “yips”: a focal dystonia of golfers. *Neurology* 39 192–195. 2915788 10.1212/wnl.39.2.192

[B55] Mesagno C. Harvey J. T. Janelle C. M. (2011). Self-presentation origins of choking: evidence from separate pressure manipulations. *J. Sport Exerc. Psychol.* 33 441–459. 10.1123/jsep.33.3.441 21659672

[B56] Mesagno C. Harvey J. T. Janelle C. M. (2012). Choking under pressure: the role of fear of negative evaluation. *Psychol. Sport Exerc.* 13 60–68. 10.1016/j.psychsport.2011.07.007

[B57] Mesagno C. Marchant D. (2013). Characteristics of polar opposites: an exploratory investigation of choking-resistant and choking-susceptible athletes. *J. Appl. Sport Psychol.* 25 72–91. 10.1080/10413200.2012.664605

[B58] Mine K. Ono K. Tanpo N. (2018). Effectiveness of management for the yips in sport. *J. Phys. Ther. Sports Med.* 2 17–25. 11132124

[B59] Muris P. Roelofs J. Rassin E. Franken I. Mayer B. (2005). Mediating effects of rumination and worry on the links between neuroticism, anxiety and depression. *Pers. Individ. Dif.* 39 1105–1111. 10.1016/j.paid.2005.04.005

[B60] Nepon T. Flett G. L. Hewitt P. L. Molnar D. S. (2011). Perfectionism, negative social feedback, and interpersonal rumination in depression and social anxiety. *Can. J. Behav. Sci.* 43:297 10.1037/a0025032

[B61] Newman M. G. Llera S. J. (2011). A novel theory of experiential avoidance in generalised anxiety disorder: a review and synthesis of research supporting a contrast avoidance model of worry. *Clin. Psychol. Rev.* 31 371–382. 10.1016/j.cpr.2011.01.008 21334285 PMC3073849

[B62] Oh I. S. Wang G. Mount M. K. (2011). Validity of observer ratings of the five-factor model of personality traits: a meta-analysis. *J. Appl. Psychol.* 96:762. 10.1037/a0021832 21142341

[B63] Philippen P. B. Lobinger B. H. (2012). Understanding the yips in golf: thoughts, feelings, and focus of attention in yips-affected golfers. *Sport Psychol.* 26 325–340. 10.1123/tsp.26.3.325

[B64] Poropat A. E. (2011). The Eysenckian personality factors and their correlations with academic performance. *Br. J. Educ. Psychol.* 81 41–58. 10.1348/000709910X497671 21391963

[B65] Prior E. E. Coates J. K. (2019). Archer’s experiences of target-panic: an interpretive phenomenological analysis. *Qual. Res. Sport Exerc. Health* 1–18. 10.1080/2159676x.2019.1599061

[B66] Rammstedt B. John O. P. (2007). Measuring personality in one minute or less: a 10-item short version of the big five inventory in English and German. *J. Res. Pers.* 41 203–212. 10.1016/j.jrp.2006.02.001

[B67] Roberts R. Rotherham M. Maynard I. Thomas O. Woodman T. (2013). Perfectionism and the yips: an initial investigation. *Sport Psychol.* 27 53–61. 10.1123/tsp.27.1.53

[B68] Roberts B. W. Smith J. Jackson J. J. Edmonds G. (2009). Compensatory conscientiousness and health in older couples. *Psychol. Sci.* 20 553–559. 10.1111/j.1467-9280.2009.02339.x 19476589 PMC2698025

[B69] Sapieja K. M. Dunn J. G. H. Holt N. L. (2011). Perfectionism and perceptions of parenting styles in male youth soccer. *J. Sport Exerc. Psychol.* 22 20–39. 10.1123/jsep.33.1.20 21451169

[B70] Schmidt N. B. Lerew D. R. Jackson R. J. (1997). The role of anxiety sensitivity in the pathogenesis of panic: prospective evaluation of spontaneous panic attacks during acute stress. *J. Abnorm. Psychol.* 106 355–364. 10.1037/0021-843x.106.3.355 9241937

[B71] Sherry S. B. Hewitt P. L. Flett G. L. Lee-Baggley D. L. Hall P. A. (2007). Trait perfectionism and perfectionistic self-presentation in personality pathology. *Pers. Individ. Diff.* 42 477–490. 10.1016/j.paid.2006.07.026

[B72] Smith M. A. Adler C. H. Crews D. Wharen R. E. Laskowski E. R. Barnes K. (2003). The ‘yips’ in golf: a continuum between a focal dystonia and choking. *Sports Med.* 33 13–31. 10.2165/00007256-200333010-00002 12477375

[B73] Smith M. A. Malo S. A. Laskowski E. R. Sabick M. Cooney W. P. Finnie S. B. (2000). A multidisciplinary study of the yips phenomenon in golf: an exploratory analysis. *Sports Med.* 30 423–437. 10.2165/00007256-200030060-00004 11132124

[B74] Sorotzkin B. (1985). The quest for perfection: avoiding guilt or avoiding shame? *Psychothe. Theory Res. Pract. Train.* 22:564 10.1037/h0085541

[B75] Stinear C. M. Coxon J. P. Fleming M. K. Lim V. K. Prapavessis H. Byblow W. D. (2006). The yips in golf: multimodel evidence for two subtypes. *Med. Sci. Sports Exerc.* 38 1980–1989. 10.1249/01.mss.0000233792.93540.10 17095933

[B76] Stoeber J. Otto K. (2006). Positive conceptions of perfectionism: approaches, evidence, challenges. *Pers. Soc. Psychol. Rev.* 10 295–319. 10.1207/s15327957pspr1004_2 17201590

[B77] Sturman E. D. (2011). Inventory subordination and its relation to personality, mood, and submissive behaviour. *Psychol. Assess.* 23 262–276. 10.1037/a0021499 21280956

[B78] Taylor S. Zvolensky M. J. Cox B. J. Deacon B. Heimberg R. G. Ledley D. R. (2007). Robust dimensions of anxiety sensitivity: development and initial validation of the anxiety Sensitivity Index-3. *Psychol. Assess.* 19:176. 10.1037/1040-3590.19.2.176 17563199

[B79] Watson D. Friend R. (1969). Measurement of social-evaluative anxiety. *J. Consult. Clin. Psychol.* 33 448–457. 10.1037/h0027806 5810590

[B80] Wilson M. (2008). From processing efficiency to attentional control: a mechanistic account of the anxiety-performance relationship. *Int. Rev. Sport Exerc. Psychol.* 1 184–201. 10.1080/17509840802400787

[B81] Woodman T. Zourbanos N. Hardy L. Beattie S. McQuillan A. (2010). Do performance strategies moderate the relationship between personality and training behaviours? An exploratory study. *J. Appl. Sport Psychol.* 22 183–197. 10.1080/10413201003664673 7622022

